# Factors Associated with Digital Health Information-Seeking Behavior Among Healthcare Professionals in Izmir: A Cross-Sectional Study

**DOI:** 10.3390/healthcare14172854

**Published:** 2026-09-04

**Authors:** Gökben Yaslı, Levent Uğurlu

**Affiliations:** 1 Department of Public Health, Faculty of Medicine, Izmir Bakircay University, 35665 Menemen, İzmir, Turkey; 2 General Surgery Clinic, Izmir Health Sciences University Tepecik Training and Research, 35020 Konak, İzmir, Turkey; dr.lugurlu@hotmail.com

**Keywords:** e-health literacy, healthcare professionals, health information-seeking behavior, ChatGPT, health information

## Abstract

**Background:** Recently, artificial intelligence-based tools such as large language models (LLMs) have further transformed health information-seeking practices. This study aimed to assess digital health information-seeking behaviors among healthcare professionals working in İzmir, Türkiye, and to examine individual and environmental factors associated with these behaviors. **Methods:** A cross-sectional study was conducted among 380 healthcare professionals in İzmir. Data were collected using an online questionnaire comprising sociodemographic and health information-seeking items and the eight-item e-health literacy scale (eHEALS). Descriptive statistics and non-parametric tests were used for unadjusted comparisons. Multivariable binary logistic regression was performed to identify factors independently associated with LLM use, while multivariable linear regression with HC3-robust standard errors was used to identify factors independently associated with e-health literacy. To reduce sparse-data instability, conceptually compatible small categories were collapsed before refitting the multivariable models. Statistical significance was set at *p* < 0.05. **Results:** Overall, 73.2% of participants reported using LLMs (e.g., ChatGPT) for health-related information seeking. After multivariable adjustment, daily internet use of 3–6 h was associated with higher odds of LLM use compared with ≤3 h/day (adjusted OR = 2.0437, 95% CI: 1.1467–3.6423, *p* = 0.0153). E-health literacy was not independently associated with LLM use (adjusted OR = 0.9875, 95% CI: 0.9599–1.0158, *p* = 0.3825). In the e-health literacy model, the combined divorced/widowed group had lower adjusted scores than married participants (B = −4.4087, 95% CI: −7.4003 to −1.4171, *p* = 0.0039). Uncertainty about institutional scientific database access was also associated with lower e-health literacy (B = −6.1805, 95% CI: −8.9255 to −3.4356, *p* < 0.0001), whereas often/always reading online health information was associated with higher scores compared with never/rarely reading it (B = 2.6264, 95% CI: 0.5112–4.7416, *p* = 0.0149). **Conclusion:** LLM use was common among healthcare professionals, but it was not independently associated with e-health literacy. Patterns of internet use, marital status, institutional database awareness, and frequency of reading online health information showed independent associations with the study outcomes. These findings support targeted digital health and AI-literacy initiatives.

## 1. Introduction

Health literacy plays a decisive role in acquiring health-related information and in using this information effectively. Therefore, it has been recognized as one of the public health objectives of the 21st century [1]. Health literacy encompasses listening and speaking skills as well as possessing various conceptual knowledge related to health [2]. The World Health Organization (WHO) defines health literacy as achieving the knowledge, personal skills, and confidence necessary to take action to improve individual and community health by modifying personal lifestyles and living conditions [3].

The internet, which has substantially transformed the accumulation of human knowledge, has also altered the nature of health-related information. With the increasing use of the internet, e-health literacy has become an important factor in promoting positive health behaviors [1]. Today, the use of internet-based health information necessitates e-health literacy. E-health literacy facilitates understanding the content of health-related information technologies and using these technologies effectively [4].

The concept of e-health literacy was defined by Norman and Skinner as “the ability to seek, find, understand, and appraise health information from electronic sources and apply the knowledge gained to addressing or solving a health problem” [5]. In addition to understanding health-related information obtained through electronic sources, e-health literacy also includes the ability to actively communicate this information to healthcare professionals [4]. The World Health Organization (WHO) describes e-health literacy as the ability to apply information acquired from electronic sources to the resolution of health-related problems [6].

E-health literacy encompasses behaviors such as using technology, thinking critically, and navigating among e-health resources. Furthermore, e-health literacy is associated with the knowledge and skills required to search for health-related information, refine searches, and distinguish scientific research from other sources [7]. Today, the emergence of the internet as a major and preferred source of health information has led to an increase in scientific studies in this field. A study analyzing data over a ten-year period demonstrated that sociodemographic factors and general health status influence online health information-seeking behavior. In addition, service providers in online health environments are required to share up-to-date information that meets individuals’ needs [8], as an increasing number of patients seek medical information via the internet.

Understanding how individuals use health information available on the internet is of critical importance. The information obtained influences individuals’ health status, their patient role, and the delivery of healthcare services [9]. In Türkiye, the information-seeking behaviors of healthcare professionals—particularly physicians and nurses—are shaped by multidimensional factors such as technological infrastructure, institutional access systems, and digital literacy. Digital platforms developed by the Ministry of Health, including e-Nabız, the Public Health Management System (HSYS), and Medula, indirectly affect access to information in clinical decision-making processes. However, because these systems primarily focus on patient data and do not provide direct access to up-to-date, evidence-based information, physicians and nurses frequently turn to international resources such as PubMed, UpToDate, and Google Scholar.

Healthcare professionals’ health information-seeking behaviors are strongly influenced by institutional infrastructure and levels of digital health literacy. Previous studies have shown that access to institutional resources plays a critical role in determining the quality and reliability of information used in clinical practice. In particular, healthcare professionals working in university hospitals generally have greater access to electronic libraries (e.g., ClinicalKey, EBSCOhost, ProQuest) and scientific databases (e.g., PubMed, Web of Science), which facilitates the use of up-to-date, evidence-based information in clinical decision-making [10]. This enhanced access may contribute to more informed clinical judgments and adherence to evidence-based practice.

In contrast, healthcare professionals employed in secondary and tertiary public hospitals often experience limited access to such institutional resources. As a result, individual information-seeking through personal mobile devices and freely accessible online sources becomes more prevalent. This reliance on non-institutional resources may increase variability in information quality and reliability. Such disparities reflect a form of institutional digital inequality, which can indirectly influence clinical decision-making processes and contribute to differences in healthcare quality across institutions [10]. Addressing these inequalities through improved institutional access and targeted digital health literacy interventions may help reduce gaps in evidence-based practice among healthcare professionals.

Previous research highlights that nurses working in rural settings are disadvantaged with regard to both internet access and digital competencies. These limitations may restrict effective engagement with digital health resources and negatively influence health information-seeking behaviors. In a systematic review conducted by Alzghaibi, it was reported that individuals with lower levels of digital health literacy spend more time searching for health information online and are at a higher risk of encountering misinformation [11]. This finding suggests that limited digital skills not only reduce efficiency in information seeking but may also compromise information accuracy and reliability.

Similarly, evidence from the Turkish context indicates that nurses predominantly fulfill their information needs through traditional sources such as clinical guidelines, procedure manuals, and face-to-face consultations with colleagues. Internet-based health information searches are generally conducted only when deemed necessary and often under time pressure. This reliance on conventional information channels may reflect both limited institutional digital infrastructure and insufficient confidence in navigating online health resources. Taken together, these findings underscore the importance of improving digital health literacy and expanding equitable access to reliable digital information systems, particularly for nurses working in resource-limited or rural healthcare settings.

From a physician’s perspective, information-seeking sources appear to vary according to area of specialization. General practitioners tend to rely on general and easily accessible resources such as Google, YouTube, and platforms provided by the General Directorate of Public Health, primarily due to time constraints and the need for rapid access to information. In contrast, specialist physicians more frequently prefer scientific and evidence-based databases, including PubMed, UpToDate, and Lexicomp [12,13,14]. This distinction reflects differences in clinical responsibilities, depth of decision-making, and the complexity of cases managed by these professional groups.

Supporting this view, findings reported by Kovarik et al. during the COVID-19 period indicated that nurses prioritized practical guidelines and concise information sources, whereas physicians predominantly consulted detailed and comprehensive literature resources [14]. Such differences can be attributed to variations in professional role definitions and decision-making mechanisms, with physicians bearing greater responsibility for diagnostic and therapeutic decisions. Consequently, these professional distinctions influence not only the choice of information sources but also the depth and rigor of information sought in clinical practice.

Recent developments indicate that digital transformation in healthcare extends beyond access to conventional online information and increasingly encompasses digitally supported education, artificial intelligence (AI), and technology-enabled healthcare practices. In health professions education, web-based and new-media teaching strategies have become increasingly integrated into learning environments. A recent network meta-analysis of 46 studies involving 6447 students demonstrated that new-media teaching approaches, including flipped classrooms, micro-classrooms, and computer-based learning, can improve several educational outcomes in pharmacology education, although their effectiveness varies according to educational background and professional field [15]. These findings highlight the importance of considering differences in professional and educational backgrounds when evaluating engagement with emerging digital technologies. At a broader healthcare-system level, AI is also becoming increasingly integrated into health-related technological innovation. Recent evidence has demonstrated expanding interactions between AI innovation, public health systems, and the transfer of health technologies, illustrating that AI-related transformation is occurring not only at the individual-user level but also across healthcare and public health ecosystems [16]. Within this rapidly evolving environment, generative AI and large language models (LLMs) represent a further change in how health information can be accessed. Unlike conventional search engines and bibliographic databases, LLMs provide conversational and rapidly generated responses to user queries. Their growing availability, however, raises important questions regarding the accuracy and reliability of AI-generated health information and users’ ability to critically evaluate such content.

Although previous studies have extensively examined e-health literacy and online health information seeking through websites, search engines, social media, and scientific databases, the emergence of LLMs requires health information-seeking behavior to be reconsidered within this changing digital environment. In particular, evidence is still developing regarding how healthcare professionals incorporate LLMs into their broader health information-seeking practices and how they perceive the reliability of the information generated by these tools. Therefore, examining conventional online information-seeking behaviors together with e-health literacy and emerging LLM-based practices may provide a more contemporary understanding of digital health information seeking among healthcare professionals.

Accordingly, this study aimed to assess digital health information-seeking behaviors and e-health literacy among healthcare professionals working in İzmir, Türkiye, and to examine their associations with selected individual and environmental characteristics. In addition, given the rapidly expanding role of generative AI in the digital health information environment, the study examined healthcare professionals’ use of LLMs for health information seeking, including frequency of use, satisfaction, perceived accuracy and reliability, and concerns regarding LLM-generated health information.

## 2. Methodology

This analytical cross-sectional study is reported with reference to the STROBE recommendations for cross-sectional studies. According to the Ministry of Health of the Republic of Türkiye’s Health Statistics Yearbook, 2023, the source population comprised 29,218 healthcare professionals working in İzmir, including 13,838 physicians, 12,656 nurses, and 2724 midwives [17]. Data were collected between 18 September and 16 October 2025.

Participants were recruited in İzmir using a non-probability voluntary response/convenience sampling approach rather than probability-based random selection. Eligible participants were healthcare professionals from the occupational groups included in the study (midwives, nurses, general practitioners, and specialist physicians) who were working in İzmir and voluntarily agreed to participate in the online survey. Because recruitment was voluntary and the number of eligible professionals who received or viewed the survey invitation was not systematically recorded, the sampling process does not ensure population representativeness and a valid response rate could not be calculated.

The target sample size was calculated for a finite source population of 29,218 using a 95% confidence level (Z = 1.96), an assumed proportion of 0.50 to reflect maximum variance, and a margin of error (d) of 0.05, using the following formula [18]. This calculation was used to define the recruitment target; it should not be interpreted as guaranteeing representativeness under the non-probability sampling design.

$$n=\frac{N\cdot Z^{2}\cdot p\cdot \left(1-p\right)}{\left(N-1)\cdot d^{2}+Z^{2}\cdot p\cdot (1-p\right)}\;n=\frac{29,218\cdot {\left(1.96\right)}^{2}\cdot 0.5\cdot 0.5}{\left(29,218-1\right)\cdot {\left(0.05\right)}^{2}+{\left(1.96\right)}^{2}\cdot 0.5\cdot 0.5}\;n\approx \frac{28,055.64}{74.00}\approx 379.2$$

Based on this calculation, the recruitment target was 380 participants, and 380 healthcare professionals were included in the analysis.

An online questionnaire was administered to 380 participants who agreed to take part in the study. The questionnaire comprised sections on personal and professional characteristics, digital and online health information-seeking behaviors, LLM use, and the e-health literacy scale (eHEALS) [19].

The e-health literacy scale (eHEALS), originally developed by Norman and Skinner, includes eight items that contribute to the e-health literacy score, in addition to two supplementary items concerning internet use that are not included in the total score. Each of the eight scored eHEALS items is rated on a five-point Likert scale from 1 (strongly disagree) to 5 (strongly agree), yielding a total score ranging from 8 to 40. The total eHEALS score is calculated by summing the responses to these eight items, with higher scores indicating higher perceived e-health literacy. The two supplementary internet-use items are not included in this total score. The Turkish adaptation by Gencer reported a Cronbach’s α of 0.886; in the present sample, Cronbach’s α for the eight scored items was 0.9768 [20].

Separately, online health information-seeking behavior was assessed using 17 categorical questions developed with reference to the U.S. National Cancer Institute’s Health Information National Trends Survey (HINTS) [19]. These 17 questions were analyzed individually and were not summed or combined into a Health Information-Seeking Behavior Scale or composite score. Thus, the eHEALS and the 17 HINTS-informed categorical questions represent two distinct components of the questionnaire. The HINTS-informed questions were not treated as a psychometric scale; therefore, internal consistency reliability was not calculated for these heterogeneous categorical items. No separate cognitive testing or pilot testing of these 17 HINTS-informed questions was conducted before the main survey.

Ethical approval for this non-interventional study was obtained from the Ethics Committee of SBÜ İzmir Tepecik Training and Research Hospital (Approval No: 2025/07-12; Date: 6 August 2025). The study was conducted in accordance with the Declaration of Helsinki. Participants were informed about the purpose of the study and confidentiality of their responses, and informed consent was obtained from each participant before participation. Participation was voluntary, and participants could withdraw at any time.

In addition to descriptive statistics, distributional normality of the eHEALS total score was assessed using the Kolmogorov–Smirnov test (D = 0.244, *p* < 0.001). For unadjusted comparisons, the Mann–Whitney U test was used for two-group comparisons and the Kruskal–Wallis H test for comparisons involving more than two groups. Spearman’s rank correlation was used for associations involving continuous variables. Bonferroni-adjusted pairwise Mann–Whitney U tests were used after a significant Kruskal–Wallis result. To identify factors independently associated with LLM use, a multivariable binary logistic regression model was fitted with LLM use (yes/no) as the dependent variable. Prespecified covariates included age, gender, profession, marital status, professional experience, chronic disease, institutional scientific database access, daily internet use, frequency of online health information seeking, and total eHEALS score. To reduce sparse-data instability and improve estimate precision, conceptually compatible categories were collapsed before refitting the primary models: nurses and midwives were combined as nursing/midwifery; divorced and widowed participants were combined as divorced/widowed; 1–5 and 6–10 years of professional experience were combined as ≤10 years; and <1 h and 1–3 h of daily internet use were combined as ≤3 h/day. Specialist physicians, married participants, ≥16 years of experience, and ≤3 h/day of internet use were used as the respective reference categories. Adjusted odds ratios (aORs), 95% confidence intervals (CIs), and *p* values are reported. A multivariable linear regression model was used for the eHEALS total score with the same collapsed profession, marital-status, professional-experience, and daily internet-use categories and with age, gender, chronic disease, institutional scientific database access, online health information-seeking frequency, frequency of reading online health information, having a preferred health-related website/social media source, and LLM use. Because the “never” reading category contained only three participants, reading frequency was also collapsed as never/rarely, sometimes, and often/always for this model. Because residual heteroskedasticity and non-normality were detected, HC3 heteroskedasticity-robust standard errors were used. No observations were missing for variables included in the primary multivariable analyses; therefore, no missing-data imputation was performed. Model fit was summarized using McFadden pseudo-R^2^ for logistic regression and R^2^/adjusted R^2^ for linear regression. Statistical significance was set at *p* < 0.05. Descriptive and non-parametric analyses were performed in IBM SPSS Statistics version 26.0, and multivariable models were verified using Python statsmodels version 0.14.6.

Additional exploratory multivariable analyses examined factors associated with LLM use frequency, satisfaction, and perceived reliability. Among the 278 participants who reported LLM use, frequency was dichotomized according to the prespecified descriptive categories as frequent use (daily/weekly/several times per week; n = 151) versus infrequent use (rarely/a few times per month; n = 127). Satisfaction was modeled as satisfied/very satisfied (n = 201) versus neutral/dissatisfied/very dissatisfied (n = 179), and perceived reliability as an unequivocal “Yes” response (n = 96) versus sometimes/no/not sure (n = 284). Binary logistic regression models included age, gender, profession, chronic disease, institutional scientific database access, daily internet use, frequency of online health information seeking, and total eHEALS score. For the LLM-frequency model, the two lowest daily internet-use categories were combined (≤3 h/day) because none of the 12 LLM users reporting <1 h/day were in the frequent-use category, which produced complete separation in the uncollapsed model. Results are reported as adjusted odds ratios (aORs), 95% confidence intervals, and *p* values.

## 3. Findings

### 3.1. Descriptive Findings

The study included 380 healthcare professionals. The mean age of participants was 44.9 years (range: 21–66), and the majority were female (76.1%). Physicians (general practitioners and specialists) constituted 57.9% of the sample, while nurses and midwives constituted 42.1%. Notably, 67.4% of the participants had 16 or more years of professional experience. The majority were married (76.3%), and 42.9% reported having a chronic illness (Table 1).

The majority of participants reported daily internet usage ranging between 3 and 6 h (40.8%), with the most frequent usage period occurring between 6:00 p.m. and midnight (49.5%). While 56.1% of respondents indicated that they had access to scientific databases at their institutions, 23.7% were unsure about such access (Table 2).

A substantial proportion (75.8%) stated that they searched for health-related information online daily or several times per week, primarily focusing on symptoms, treatment options, and medications. The sources used for these searches included academic databases (e.g., PubMed, Scopus) as well as large language models (LLMs) such as ChatGPT.

When examining the use of LLMs for health information retrieval, it was found that 73.2% of participants had utilized such platforms, whereas 26.8% had not. Among the users, 54.3% reported using LLMs “daily, weekly, or several times per week,” while 45.7% indicated “occasional or monthly” use. Regarding satisfaction with the health information obtained from LLMs, 52.9% of respondents expressed being “satisfied or very satisfied,” 39.7% were “neutral,” and 7.8% reported being “dissatisfied or very dissatisfied.”

An analysis of participants’ trust in online health and medical information revealed that 71.3% reported a moderate level of trust, 16.8% expressed low trust, 7.9% had high trust, 3.2% did not trust at all, and only 0.8% indicated complete trust (Table 3).

Regarding sources of health-related information, 74.7% of the participants reported primarily using the internet, while 16.3% preferred books, and 9.0% relied on other sources such as colleagues, television, or printed brochures.

A significant proportion (88.9%) indicated that they had searched for health information on behalf of others, such as family members or acquaintances, within the past 12 months. Additionally, 85.5% reported searching online for information about healthcare institutions, 68.4% had scheduled a medical appointment online, and 43.9% had purchased a health product online during the same period.

In the past year, 92.4% had searched for information about medications online, whereas 7.6% had not. In terms of time spent seeking health information on the internet, 67.1% reported spending less than one hour, 23.9% between 1 and 2 h, 3.4% exactly 2 h, and 5.5% more than 2 h.

When asked about the frequency of reading online health information, 36.3% reported doing so occasionally, 31.3% frequently, 23.9% rarely, 7.6% always, and 0.8% never. Additionally, 42.1% of participants indicated that they had **a** preferred health website or social media account, such as hospital websites, physicians’ pages, or Instagram/Twitter accounts, while 57.9% reported no such preference.

Regarding the use of the internet for accessing diagnostic results, 76.6% stated that they had used it to view laboratory or radiology reports, while 23.4% had not. Social media platforms were widely used for health-related content, with 62.9% reporting that they had watched videos related to health or disease on platforms such as Instagram, Facebook, or Twitter, and 54.7% on YouTube.

Overall, the majority of participants (92.4%) had conducted online searches for medications in the past 12 months, and 88.9% had sought health information on behalf of others. Additionally, 76.6% used the internet to access their diagnostic test results, reflecting a high level of engagement with digital health resources.

The mean score for the item “I know which health resources are accessible online” was 3.39 ± 1.22, while the item “I know where to find useful health resources on the internet” had a mean of 3.47 ± 1.19. Similarly, participants reported a mean score of 3.50 ± 1.22 for “I know how to find useful health resources on the internet” and 3.55 ± 1.23 for “I know how to use the internet to find answers to health-related questions” (Table 4).

The item “I know how to use health information I find online to help me” received a mean score of 3.58 ± 1.22. Furthermore, the item “I have the skills to evaluate the health resources I find on the internet” had a mean of 3.65 ± 1.25. The highest mean score (3.71 ± 1.22) was reported for the item “I can distinguish between high-quality and low-quality online health resources.”

Finally, the statement “I feel confident in using online information when making health-related decisions” was rated at a mean of 3.59 ± 1.24, indicating a moderate-to-high level of self-efficacy in applying e-health information to decision-making.

### 3.2. Inferential Findings

When the relationship between participants’ e-health literacy scores and sociodemographic characteristics was examined, no statistically significant Spearman correlation was found between age and e-health literacy (r_s = −0.0628, *p* = 0.2221).

In terms of gender, the mean eHEALS score was 28.72 ± 8.40 for women and 27.73 ± 11.08 for men. The difference was not statistically significant (U = 11,846.5000, *p* = 0.1470).

When evaluated by professional group, the mean scores were 27.75 ± 8.10 for midwives, 29.09 ± 7.00 for nurses, 28.18 ± 10.25 for general practitioners, and 28.34 ± 10.30 for specialist physicians. The differences between these groups were not statistically significant (H = 3.9630, *p* = 0.2655) (Table 5).

When marital status was considered, the average scores were 29.24 ± 7.93 for single participants, 28.93 ± 8.94 for married participants, 24.88 ± 11.12 for divorced participants, and 21.50 ± 7.12 for widowed participants. The overall difference was statistically significant (H = 9.4928, *p* = 0.0234). Bonferroni-adjusted pairwise Mann–Whitney U tests showed lower scores among widowed participants compared with single participants (adjusted *p* = 0.0237) and married participants (adjusted *p* = 0.0437); other pairwise comparisons were not statistically significant.

Based on years of professional experience, the mean scores were 25.87 ± 8.89 for those with 1–5 years, 30.12 ± 9.04 for 6–10 years, 29.53 ± 8.44 for 11–15 years, and 28.12 ± 9.29 for those with 16 years or more of experience. No statistically significant difference was found (H = 4.9102, *p* = 0.1785).

Finally, regarding the presence of chronic illness, the mean score was 28.97 ± 9.35 among those with a chronic condition and 28.11 ± 8.92 among those without. The difference was not statistically significant (U = 16,582.5000, *p* = 0.2899).

When examining the relationship between participants’ e-health literacy scores and their online health information-seeking behaviors, significant differences were observed in certain variables.

The majority of participants reported a moderate level of trust in online health and medical information (71.3%) and stated that they primarily accessed health-related information through internet sources (74.7%). There was no statistically significant difference in e-health literacy scores between those who searched for health information online for others and those who did not (U = 5999.5000, *p* = 0.0961). Similarly, no significant differences were found for searching for healthcare institutions online (U = 7783.0000, *p* = 0.1191), making a doctor’s appointment online (U = 15,586.5000, *p* = 0.9894), purchasing medication or dietary supplements online (U = 17,462.5000, *p* = 0.7575), or searching for medication information online (U = 4658.0000, *p* = 0.4405) (Table 6).

There was no significant difference according to time spent searching for health information online (H = 2.5345, *p* = 0.2816). However, e-health literacy differed significantly according to the frequency of reading online health information (H = 21.6813, *p* = 0.0002).

Participants with a preferred health-related website or social media account also differed significantly in e-health literacy (U = 14,561.5000, *p* = 0.0035). This unadjusted association was subsequently evaluated in the multivariable model.

Conversely, no statistically significant differences were found for using the internet to view laboratory or radiological results (U = 12,244.5000, *p* = 0.4293), watching health-related videos on social media (U = 16,169.5000, *p* = 0.5039), or watching such videos on YouTube (U = 16,834.0000, *p* = 0.3146).

### 3.3. Multivariable Regression Analyses

After collapsing sparse categories and refitting the model, the multivariable binary logistic regression model for LLM use remained statistically significant overall (likelihood-ratio *p* = 0.0254; McFadden pseudo-R^2^ = 0.0651). After adjustment, participants reporting 3–6 h of daily internet use had higher odds of LLM use than those reporting ≤3 h/day (aOR = 2.0437, 95% CI: 1.1467–3.6423, *p* = 0.0153). No independent association was observed between total eHEALS score and LLM use (aOR = 0.9875, 95% CI: 0.9599–1.0158, *p* = 0.3825). Other covariates did not reach the conventional 0.05 significance threshold (Table 7).

After refitting with the collapsed categories, the multivariable e-health literacy model remained statistically significant overall (R^2^ = 0.1774; adjusted R^2^ = 0.1340; robust model *p* < 0.0001). Compared with married participants, the combined divorced/widowed group had lower adjusted eHEALS scores (B = −4.4087, 95% CI: −7.4003 to −1.4171, *p* = 0.0039). Participants who were unsure whether their institution provided access to scientific databases also had lower adjusted eHEALS scores than those reporting no access (B = −6.1805, 95% CI: −8.9255 to −3.4356, *p* < 0.0001). Often/always reading online health information was associated with higher eHEALS scores than never/rarely reading such information (B = 2.6264, 95% CI: 0.5112–4.7416, *p* = 0.0149). LLM use itself was not independently associated with eHEALS score (B = −1.0560, 95% CI: −2.9840 to 0.8720, *p* = 0.2831) (Table 8).

Profession-based subgroup analyses showed that LLM use differed significantly across occupational groups (χ^2^(3) = 13.8407, *p* = 0.0031). LLM use was reported by 33 of 36 midwives (91.6667%), 87 of 124 nurses (70.1613%), 69 of 108 general practitioners (63.8889%), and 89 of 112 specialist physicians (79.4643%). After Holm adjustment for multiple pairwise comparisons, LLM use remained significantly higher among midwives than among nurses (adjusted *p* = 0.0414) and general practitioners (adjusted *p* = 0.0075), and higher among specialist physicians than among general practitioners (adjusted *p* = 0.0448). No other pairwise comparisons remained significant. In contrast, e-health literacy scores did not differ significantly across professional groups (Kruskal–Wallis H = 3.9630, *p* = 0.2655). Mean ± SD eHEALS scores were 27.7500 ± 8.1042 for midwives, 29.0887 ± 7.0029 for nurses, 28.1759 ± 10.2536 for general practitioners, and 28.3393 ± 10.3005 for specialist physicians (Table 9).

Regarding perceived risks of LLM use, 184 participants (48.4211%) identified misleading or inaccurate information as their primary concern, 98 (25.7895%) were concerned about generic responses that may not adequately account for individual circumstances, 50 (13.1579%) reported privacy/anonymity concerns, and 33 (8.6842%) expressed concern about excessive reliance on AI-based applications. General online health information verification practices were also common: 262 participants reported checking multiple reliable sources and 237 reported consulting healthcare professionals, whereas 17 reported not verifying online health information. These verification items referred to online health information in general and were not specific to LLM-generated content (Table 10).

## 4. Discussion

Advances in science and technology, together with increased internet access, have led individuals to become more dependent on information and have enabled rapid and easy access to all types of information online. In the present study, variables such as age, gender, profession, and years of professional experience were found to have no statistically significant effect on e-health literacy. This finding is noteworthy, as it suggests that healthcare professionals have reached comparable levels of digital competence regardless of their age or professional background.

In contrast, e-health literacy differed significantly across marital-status groups in the unadjusted analysis. Bonferroni-adjusted pairwise comparisons indicated lower scores among widowed participants compared with married and single participants; however, the widowed subgroup was very small (n = 6), so these unadjusted comparisons should be interpreted cautiously. To reduce sparse-data instability in the multivariable analysis, divorced and widowed participants were combined into a single category. The combined divorced/widowed group had lower adjusted eHEALS scores than married participants. This finding should be interpreted as an association at the level of the combined category rather than as evidence of separate effects for divorced and widowed participants.

The marital-status association may reflect differences in social context or support, but these mechanisms were not directly measured in the present study. Yang et al. (2017) [1] reported that social support is positively associated with e-health literacy and health-promoting lifestyle behaviors, while the framework proposed by Norman and Skinner emphasizes that e-health literacy involves cognitive and social as well as technical competencies [5]. Accordingly, the present marital-status finding should be regarded as hypothesis-generating rather than causal.

In the present study, participants were found to engage in online information-seeking behaviors particularly during after-work hours. While 56.1% of the participants reported having access to scientific databases within their institutions, 23.7% stated that they were not aware of whether such access was available. Institutional access to scientific databases is generally more limited for healthcare professionals working in secondary and tertiary public hospitals. As a result of these limitations, staff are often required to conduct individual searches using personal mobile devices to meet their professional information needs. This finding indicates a need for better promotion of institutional scientific database access and increased informational support for employees.

The digital hospital status of an institution, the availability of technological infrastructure, access to scientific databases, and an organizational culture that supports digitalization constitute key external factors that facilitate the development of employees’ digital competencies. Conversely, time constraints and access barriers emerge as major obstacles that hinder the effective use of e-health literacy. Studies have shown that individuals who frequently search public institution websites and health portals are more competent in evaluating the reliability of health information and tend to have higher levels of e-health literacy. Accordingly, the importance of programs aimed at improving digital health literacy is emphasized as a critical component of public health strategies [21,22,23].

A total of 75.8% of the participants reported searching for online health information either daily or several times a week. The most frequently searched topics included symptoms, treatment options, and medication-related information. The most used platforms were academic and artificial intelligence-based resources such as Google Scholar, PubMed, Scopus, Web of Science, and ChatGPT. Physicians, in particular, tend to prefer scientific databases (e.g., PubMed, UpToDate, Lexicomp), professional journals, and official websites of health authorities rather than general sources such as television, radio, or social media when seeking medical information.

Regular use of such evidence-based and professional resources enhances physicians’ competencies across multiple dimensions of health literacy, including searching for, locating, evaluating, and ethically using health information. Moreover, tools such as UpToDate and PubMed support clinical decision-making by reducing reliance on intuitive approaches and enabling faster and safer access to evidence-based information, thereby contributing to improved clinical practice [24].

In our study, analysis of responses to the question “How often have you read health-related information in the past 12 months?” revealed that individuals who read health information more frequently had significantly higher e-health literacy scores. This finding indicates that increased engagement with digital information contributes to greater awareness and competence. As the frequency of reading health information increases, individuals’ abilities to evaluate, discriminate, and apply information also improve. This result is consistent with the findings of Li et al., who reported that online health information-seeking behaviors can enhance individuals’ knowledge levels over time [25].

Today, the internet has become the most widely used primary source of information for conducting health-related research and obtaining health information, both among the general public and healthcare professionals. According to data from the Turkish Statistical Institute (TURKSTAT), the proportion of internet users in Türkiye who searched for health-related information increased from 69.3% in 2019 to 87.1% in 2023 [17]. Internet use is defined as the third most popular online activity, following email use and pre-purchase product research. However, the use of the internet as an information source also entails critical risks, including:

A substantial proportion of health-related information available on the internet has been found to be non-evidence-based, lacking expert opinion, and outdated. Incorrect or misleading information may direct individuals toward inappropriate diagnoses, treatments, and health behaviors [26]. In the present study, the internet emerged as the primary source of health information, with 74.7% of participants reporting that they primarily obtained health-related information online. In contrast, the use of books and other printed sources was relatively low. This finding clearly demonstrates the central role of digitalization in accessing health information.

In the present study, 73.2% of participants reported using large language models such as ChatGPT to search for health-related information, and 52.9% indicated that they were satisfied with these platforms. This finding suggests that artificial intelligence-based tools have been widely adopted among healthcare professionals. However, only 25.3% of participants considered these sources to be “accurate and reliable,” while 52.1% reported that they found the information to be “sometimes accurate.” The coexistence of high usage rates and relatively low levels of trust represents an unexpected finding and reflects a pragmatic approach among healthcare professionals toward rapid access to information despite concerns regarding reliability. This pattern highlights ongoing trust issues associated with AI-supported systems and underscores the need for further improvement in information accuracy and validation mechanisms [27]. In addition, a statistically significant and positive relationship was identified between digital health literacy and artificial intelligence literacy. Previous research suggests that AI literacy functions as a catalyst that supports and enhances digital health literacy. To ensure the effective implementation of digital transformation in the healthcare sector, it is recommended that these two competencies be developed in a coordinated and integrated manner among healthcare professionals [26].

In the present study, 88.9% of participants reported searching for online health information for their relatives within the past 12 months, while 68.4% stated that they had researched a healthcare institution online. In addition, 92.4% reported seeking online information related to medications. These findings indicate that the internet has become a primary source, particularly for accessing pharmaceutical information. Today, the internet is the most important and widely used resource for conducting health-related research and obtaining health information among both the general population and healthcare professionals. The rapid, easy, free, and anonymous access to information has made the internet an indispensable tool in individuals’ efforts to understand and manage their health conditions [28]. The finding that users’ orientation toward specific websites or social media platforms was statistically significant supports the observations of Pan et al., who emphasized that individuals’ preferences for information sources influence their perceptions of information quality and levels of trust. Pan and colleagues reported that users tend to place greater trust in hospital websites and pages featuring expert opinions and that reliance on such sources can directly affect health-related behaviors [29].

On the other hand, the lack of a statistically significant association between behaviors such as watching health-related videos on platforms like YouTube and Instagram may be explained by the “information quality problem” highlighted by Jung and Lee (2022) [24]. Jung and Lee noted that the reliability of health information shared on social media platforms is generally low and that users tend to consume such information in a largely passive manner. This passive consumption, combined with questionable content quality, may explain why these behaviors did not yield statistically significant results in the present study.

Participants reported spending less than one hour when searching for health information, indicating a strong tendency toward rapid access to information. Reading frequency serves as an important indicator of individuals’ level of engagement with information and can be directly associated with e-health literacy. Daily internet use and the extent to which individuals follow developments in digital technologies are among the critical factors determining literacy levels. In addition, the age at which individuals begin using smart devices has been shown to be particularly influential in dimensions related to professional productivity and social interaction [23]. Following 2020, the concept of digital health literacy has gained increasing prominence in the literature. Notably, it has been emphasized that the frequency of reading health information reflects digital literacy and that individuals who engage more frequently with such information demonstrate higher levels of information awareness. Consistent with these findings, our study revealed that individuals who regularly read health-related information online differed significantly in terms of e-health literacy levels (*p* < 0.001), directly aligning with Tran et al.’s results [30]. Individuals who regularly use the internet to read health information appear to evaluate such information more consciously and actively, and they tend to prefer reliable sources or favored platforms. This preference may significantly influence how health information is used. Participants who read health information more frequently demonstrated significantly higher levels of e-health literacy, suggesting that increased interaction with digital information enhances awareness and gradually improves knowledge levels. Furthermore, healthcare professionals who consistently follow reliable and verified sources—such as hospital websites or expert physician accounts—exhibited higher e-health literacy levels. This finding indicates that “selective information-seeking” behavior positively contributes to digital health competence. With the COVID-19 pandemic, the use of the internet as a source of health information increased dramatically, substantially amplifying the role of digital resources in individuals’ health information-seeking, decision-making, and behavior development processes [28]. This interpretation is consistent with prior evidence showing that online health information-seeking patterns and appraisal practices are related to e-health literacy [25].

Participants reported the existence of various health-related content platforms, and the widespread behavior of watching health-related videos on social media was particularly notable. This finding highlights the important role of visual and short-form content in digital information consumption. Given that healthcare professionals often work under significant time pressure, presenting complex evidence syntheses through visual summaries and short videos is considered an effective tool for facilitating the translation of knowledge into clinical practice [24].

In this study, the relationships between participants’ total e-health literacy scores and selected key demographic variables were also examined. The mean scores across different professional groups were similar, and no statistically significant differences were observed. Although nurses demonstrated the highest mean score, this difference did not reach statistical significance. These findings indicate that professional title alone does not have a determining effect on e-health literacy levels. However, it was observed that nurses, particularly during crisis periods, require greater access to practical training and visual educational resources (e.g., videos and webinars) to sustain patient care effectively [24].

A particularly relevant finding of this study was the integration of LLMs into healthcare professionals’ health information-seeking practices. Overall, 73.2% of participants reported using LLMs such as ChatGPT for health-related information; however, only 25.3% considered the information accurate and reliable, while 52.1% considered it reliable only sometimes. Misleading or inaccurate information was also the most frequently reported concern. Thus, widespread use should not be interpreted as equivalent to trust.

These findings should be considered within the broader digital transformation of healthcare. Recent evidence indicates increasing integration of digital technologies into health profession education [15] and growing interaction between AI innovation, public health, and health-technology development [16]. The present study extends this perspective by examining LLM use alongside e-health literacy and conventional online health information-seeking behaviors among practicing healthcare professionals.

After collapsing sparse categories and refitting the multivariable models, 3–6 h of daily internet use remained independently associated with LLM use compared with ≤3 h/day, whereas e-health literacy was not. The revised confidence interval was narrower than under the original sparse reference categorization, supporting improved estimate precision. In the e-health literacy model, the combined divorced/widowed group and participants who were unsure about institutional scientific database access had lower adjusted scores, while often/always reading online health information was associated with higher e-health literacy than never/rarely reading it. These findings indicate associations rather than causal relationships, and the direction of the relationship between online information seeking and e-health literacy cannot be established.

Profession-based analyses also indicated differences in LLM adoption despite broadly comparable e-health literacy levels. These findings may reflect differences in professional information needs or digital practices; however, they should be interpreted cautiously because the comparisons were observational and some professional subgroups were relatively small.

Participants’ concerns about LLMs included inaccurate or misleading information, insufficiently individualized responses, privacy concerns, and excessive reliance on AI. However, awareness of hallucinations, algorithmic bias, broader ethical issues, and the specific need for human verification was not directly assessed. LLM-generated health information should therefore be regarded as supportive rather than autonomous clinical evidence and verified against authoritative evidence-based sources and professional judgment.

This distinction is particularly important because LLMs differ from established professional resources such as PubMed, UpToDate, clinical guidelines, institutional databases, Google Scholar, and medical textbooks. LLMs can provide rapid and synthesized responses, whereas established resources generally offer more transparent access to identifiable evidence. Accordingly, LLMs may be better regarded as complementary rather than replacement sources in clinical information seeking.

Digital competence also extends beyond e-health literacy and may include AI literacy, digital self-efficacy, critical appraisal, cybersecurity awareness, and evidence-based information skills. Because these domains were not measured using dedicated validated instruments, participants’ self-reported verification behaviors should not be interpreted as validated measures of broader digital competence.

Institutional access to scientific resources represents another important finding. Although 56.1% of participants reported institutional access to scientific databases, 23.7% were unsure whether such access was available. This uncertainty may reflect limited awareness of existing resources, although the underlying reasons were not directly assessed. Institutions should therefore not only provide access to scientific databases but also communicate available resources clearly and support healthcare professionals in evidence retrieval and database use.

More broadly, differences in access to scientific databases, digital infrastructure, training, and awareness may contribute to institutional digital inequality. This issue becomes increasingly relevant with growing LLM use because access to authoritative evidence is essential for verifying AI-generated information. Healthcare institutions should therefore combine equitable access to scientific resources with training in digital and AI literacy, critical appraisal, privacy, and responsible LLM use.

Finally, the marital-status finding requires particular caution. The widowed subgroup included only six participants (1.6%), and although the overall comparison was statistically significant, the effect size was small (ε^2^ = 0.0173, 95% CI: 0.0073–0.0513). This finding should therefore be considered exploratory and requires confirmation in larger and more balanced samples.

### Limitations

This study has several limitations. First, its cross-sectional design precludes conclusions about temporal ordering or causality, and reverse causality remains possible. Second, data were collected using self-reported online questionnaires and may be affected by recall, reporting, and social desirability biases.

Recruitment was based on a non-probability voluntary response/convenience sampling approach, which may have overrepresented healthcare professionals with greater digital engagement or interest in emerging technologies. Because the number of eligible professionals who received or viewed the survey invitation was not systematically recorded, a valid response rate could not be calculated; information on non-respondents was unavailable, and responder–non-responder comparisons could not be performed. These unavailable recruitment data limit assessment of non-response bias. The study was also limited to İzmir and included midwives, nurses, general practitioners, and specialist physicians but not pharmacists, dentists, or other allied healthcare professionals. Therefore, the findings should not be regarded as population-level estimates for all healthcare professionals in İzmir or Türkiye.

The 17 HINTS-informed categorical questions were not subjected to formal cross-cultural validation, cognitive interviewing, or pilot testing in the present population. Dedicated validated instruments were also not used to assess AI literacy, digital self-efficacy, critical appraisal, cybersecurity awareness, evidence-based information skills, or specific awareness of LLM-related risks. Furthermore, traditional information sources and LLMs were not assessed using directly comparable measures; therefore, conclusions about whether LLMs complement or replace established evidence-based resources cannot be drawn.

To reduce sparse-data instability in the multivariable analyses, conceptually compatible categories were collapsed for profession, marital status, professional experience, daily internet use, and, in the eHEALS model, online health information reading frequency. This reduced reliance on very small categories and improved the precision of several estimates. Nevertheless, category collapsing trades detail for stability and may mask heterogeneity within the combined groups. In particular, the unadjusted widowed subgroup remained very small (n = 6; 1.6%), so findings involving that original subgroup should be regarded as exploratory. Point estimates should be interpreted together with their 95% confidence intervals, and non-significant findings should not be interpreted as evidence of equivalence or absence of an association.

Future studies should use larger, multicenter and professionally diverse samples, preferably with probability-based or stratified sampling and documented response rates. Longitudinal designs, validated multidimensional measures, and direct comparisons between LLMs and established evidence-based resources would further clarify changes in e-health literacy, LLM adoption, verification behaviors, and safe generative AI use.

## 5. Conclusions

This study examined e-health literacy, online health information-seeking behaviors, and LLM use among healthcare professionals. After collapsing sparse categories and refitting the multivariable models, 3–6 h of daily internet use remained independently associated with LLM use compared with ≤3 h/day, whereas e-health literacy was not. Lower adjusted e-health literacy scores were observed in the combined divorced/widowed group and among participants who were unsure about institutional scientific database access, while often/always reading online health information was associated with higher e-health literacy than never/rarely reading it.

Overall, the findings demonstrate substantial engagement with digital health information and widespread LLM use, accompanied by cautious perceptions of LLM reliability. Healthcare institutions should support access to authoritative scientific resources, strengthen digital and AI literacy, and promote critical verification of AI-generated health information against evidence-based sources and professional judgment.

## Figures and Tables

**Table 1:** Demographic characteristics of the participants.

Variable	n	%
**Gender**		
Female	289	76.1%
Male	91	23.9%
**Profession**		
Specialist physician	112	29.5%
General practitioner	108	28.4%
Nurse	124	32.6%
Midwife	36	9.5%
**Type of Institution**		
Family Health Center	19	5.0%
Municipality	6	1.6%
State Hospital	88	23.2%
Retired	3	0.8%
Public Health Laboratory	6	1.6%
Provincial Health Directorate	105	27.6%
District Health Directorate	27	7.1%
Private Hospital	12	3.2%
Community Health House	3	0.8%
Healthy Life Center	8	2.1%
Social Security Institution (SGK)	21	5.5%
University Hospital	82	21.6%
**Marital Status**		
Single	49	12.9%
Divorced	35	9.2%
Widowed	6	1.6%
Married	290	76.3%
**Years of Experience**		
1–5 years	16	4.2%
6–10 years	32	8.4%
11–15 years	76	20.0%
16 years and above	256	67.4%
**Presence of Chronic Illness**		
No	217	57.1%
Yes	163	42.9%

**Table 2:** Internet usage and use of large language models among participants.

Item	n	%
**Average daily internet use (via computer, tablet, or phone)**		
Less than 1 h	22	5.8%
1–3 h	110	28.9%
3–6 h	155	40.8%
More than 6 h	93	24.5%
**Most frequent time of day for internet use**		
06:00–12:00	28	7.4%
12:00–18:00	164	43.2%
18:00–24:00	188	49.5%
**Access to scientific databases at institution**		
No	77	20.3%
Yes	213	56.1%
Not sure	90	23.7%
**Frequency of searching for health-related information online**		
Rarely or never	40	10.5%
Daily or several times a week	288	75.8%
Weekly or several times a month	52	13.7%
**Main topics searched online (multiple responses)**		
Symptoms and diseases	241	63.4%
Treatment options	231	60.8%
Medications and side effects	198	52.1%
Current articles	144	37.9%
Nutrition and diet advice	108	28.4%
Exercise and fitness recommendations	103	27.1%
Mental health issues	52	13.7%
**Platforms used for health information (multiple responses)**		
Google Scholar, PubMed, Scopus, Web of Science, etc.	326	85.8%
AI applications (e.g., ChatGPT)	192	50.5%
Dergipark, TR Dizin, YÖK Thesis Center, etc.	107	28.2%
UpToDate, ClinicalKey, etc.	73	19.2%
Social media platforms	46	12.1%
Chatbots (e.g., ChatGPT)	18	4.7%
**Confidence in accuracy of online health information**		
Not confident/Not at all confident	50	13.2%
Neutral	79	20.8%
Somewhat confident	211	55.5%
Very confident	40	10.5%
**How participants verify online health information (multiple responses)**		
Checking multiple reliable sources	262	68.9%
Consulting health professionals	237	62.4%
Considering information provided by AI applications	111	29.2%
Discussing with family members or friends	30	7.9%
I do not verify the information	17	4.5%
**Have you ever used LLMs (e.g., ChatGPT) to search for health info?**		
Yes	278	73.2%
No	102	26.8%
**If yes, how often do you use LLMs for health information?**		
Rarely/A few times a month	127	45.7%
Daily/Weekly/Several times a week	151	54.3%
**Satisfaction with health information from LLMs**		
Satisfied/Very satisfied	201	52.9%
Neutral	151	39.7%
Dissatisfied/Very dissatisfied	28	7.4%
**Do you find LLMs’ health information accurate and reliable?**		
Yes	96	25.3%
No	40	10.5%
Sometimes	198	52.1%
Not sure	46	12.1%
**Concerns about using LLMs for health information**		
Risk of misleading/inaccurate information	184	48.4%
Generic responses that ignore individual context	98	25.8%
**Can LLMs be used for more comprehensive health consultation?**		
Yes	169	44.5%
No	211	55.5%

Note: Percentages for multiple-response items are calculated using N = 380 as the denominator and therefore may sum to more than 100%. LLM use frequency percentages are calculated among LLM users (n = 278).

**Table 3:** Participants’ online health information-seeking behaviors.

Question	Response	n	%
1. How much do you generally trust health and medical information found online?	Not at all	12	3.2%
	Slightly	64	16.8%
	Moderately	271	71.3%
	Very much	30	7.9%
	Completely	3	0.8%
2. What is your primary source of information when seeking details about a disease?	Internet	284	74.7%
	Books	62	16.3%
	Other	34	9.0%
3. In the past 12 months, have you searched the internet for health information for others (e.g., family/friends)?	Yes	338	88.9%
	No	42	11.1%
4. In the past 12 months, have you searched for any healthcare institution online?	Yes	325	85.5%
	No	55	14.5%
5. In the past 12 months, have you scheduled a doctor’s appointment online?	Yes	260	68.4%
	No	120	31.6%
6. In the past 12 months, have you purchased any health products (e.g., medication, supplements) online?	Yes	167	43.9%
	No	213	56.1%
7. In the past 12 months, have you searched for information about any medication online?	Yes	351	92.4%
	No	29	7.6%
8. How much time do you typically spend searching for health information online?	Less than 1 h	255	67.1%
	1–2 h	91	23.9%
	Exactly 2 h	13	3.4%
	More than 2 h	21	5.5%
9. How often have you read online health information in the past 12 months?	Never	3	0.8%
	Rarely	91	23.9%
	Sometimes	138	36.3%
	Often	119	31.3%
	Always	29	7.6%
10. Do you have any favorite websites or sources for health-related information (e.g., hospital websites, physician blogs, Instagram, Twitter)?	Yes	160	42.1%
	No	220	57.9%
11. In the past 12 months, have you used the internet to view lab or radiological results?	Yes	291	76.6%
	No	89	23.4%
12. In the past 12 months, have you watched a health or disease-related video on social media (e.g., Instagram, Facebook, Twitter)?	Yes	239	62.9%
	No	141	37.1%
13. In the past 12 months, have you watched a health-related video on YouTube?	Yes	208	54.7%
	No	172	45.3%

**Table 4:** Descriptive statistics of e-health literacy scale items.

Item	$\bar{X}$	SD
1. I know which health resources are accessible online.	3.39	1.22
2. I know where to find useful health resources on the internet.	3.47	1.19
3. I know how to find useful health resources on the internet.	3.50	1.22
4. I know how to use the internet to find answers to health-related questions.	3.55	1.23
5. I know how to use health information I find online to help me.	3.58	1.22
6. I have the skills to evaluate the health resources I find on the internet.	3.65	1.25
7. I can distinguish between high-quality and low-quality online health resources.	3.71	1.22
8. I feel confident in using online information when making health-related decisions.	3.59	1.24

**Table 5:** Comparison of E-Health Literacy Scores according to demographic variables.

Variable	Mean ± SD	Test Statistic	*p*-Value	Effect Size (95% CI)
**Age**	44.94 ± 8.65 (years)	r_s = −0.0628	0.2221	r_s_ = −0.0628 (−0.1659 to 0.0419)
**Gender**		U = 11,846.5000	0.1470	r_rb = −0.0991 (−0.2573 to 0.0591)
Female	28.72 ± 8.40			
Male	27.73 ± 11.08			
**Profession**		H = 3.9630	0.2655	ε^2^ = 0.0026 (0.0000 to 0.0404)
Midwife	27.75 ± 8.10			
Nurse	29.09 ± 7.00			
General Practitioner	28.18 ± 10.25			
Specialist	28.34 ± 10.30			
**Marital Status**		H = 9.4928	0.0234	ε^2^ = 0.0173 (0.0073 to 0.0513)
Single	29.24 ± 7.93			
Married	28.93 ± 8.94			
Divorced	24.88 ± 11.12			
Widowed	21.50 ± 7.12			
**Professional Experience**		H = 4.9102	0.1785	ε^2^ = 0.0051 (0.0000 to 0.0419)
1–5 years	25.87 ± 8.89			
6–10 years	30.12 ± 9.04			
11–15 years	29.53 ± 8.44			
16 years and above	28.12 ± 9.29			
**Presence of Chronic Disease**		U = 16,582.5000	0.2899	r_rb = 0.0624 (−0.0593 to 0.1757)
Yes	28.97 ± 9.35			
No	28.11 ± 8.92			

Note: r_s_, Spearman’s rank correlation coefficient; r_rb, rank-biserial correlation coefficient; ε^2^, epsilon-squared effect size; U, Mann–Whitney U statistic; H, Kruskal–Wallis H statistic; CI, confidence interval.

**Table 6:** Comparison of e-health literacy scale scores based on online health information-seeking behaviors.

Behavior	Mean ± SD/n (%)	U/H Statistic	*p*-Value	Effect Size (95% CI)
1. Overall, how much do you trust health and medical information on the internet?	Moderate (n = 271; 71.3%)	–	–	
2. What is your primary source of information about a disease?	All internet sources (n = 284; 74.7%)	–	–	
3. In the past 12 months, have you searched for health information online for someone else?	Yes	U = 5999.5000	0.0961	r_rb = 0.1548 (−0.0242 to 0.3287)
	No			
4. In the past 12 months, have you searched for a healthcare institution online?	Yes	U = 7783.0000	0.1191	r_rb = 0.1292 (−0.0157 to 0.2669)
	No			
5. In the past 12 months, have you made a medical appointment online?	Yes	U = 15,586.5000	0.9894	r_rb = 0.0009 (−0.1200 to 0.1280)
	No			
6. In the past 12 months, have you purchased any health products (e.g., medication, supplements) online?	Yes	U = 17,462.5000	0.7575	r_rb = 0.0182 (−0.0925 to 0.1326)
	No			
7. In the past 12 months, have you searched for information about any medication online?	Yes	U = 4658.0000	0.4405	r_rb = 0.0848 (−0.0680 to 0.2409)
	No			
8. How much time do you spend searching for health information online?	<1 h/1–2 h/2 h/>2 h	H = 2.5345	0.2816	ε^2^ = 0.0014 (0.0000 to 0.0328)
9. How often have you read health information online in the past 12 months?	Never/Rarely/Sometimes/Usually/Always	H = 21.6813	0.0002	ε^2^ = 0.0472 (0.0189 to 0.1038)
10. Do you have a favorite health-related website or social media account? (e.g., hospital, Instagram)	Yes	U = 14,561.5000	0.0035	r_rb = 0.1726 (0.0601 to 0.2855)
	No			
11. In the past 12 months, have you used the internet to view lab or radiology results?	Yes	U = 12,244.5000	0.4293	r_rb = 0.0544 (−0.0792 to 0.1880)
	No			
12. In the past 12 months, have you watched any health-related videos on social media?	Yes	U = 16,169.5000	0.5039	r_rb = 0.0404 (−0.0773 to 0.1600)
	No			
13. In the past 12 months, have you watched any health-related videos on YouTube?	Yes	U = 16,834.0000	0.3146	r_rb = 0.0589 (−0.0570 to 0.1732)
	No			

Note: r_rb, rank-biserial correlation coefficient; ε^2^, epsilon-squared effect size; U, Mann–Whitney U statistic; H, Kruskal–Wallis H statistic; CI, confidence interval.

**Table 7:** Multivariable binary logistic regression analysis of factors associated with LLM use.

Predictor	aOR	95% CI	*p* Value
Age (per year)	1.0017	0.9522–1.0538	0.9485
Male (ref: female)	0.9674	0.5316–1.7606	0.9137
Nursing/midwifery (ref: specialist physician)	0.8078	0.4132–1.5792	0.5326
General practitioner (ref: specialist physician)	0.5084	0.2581–1.0016	0.0505
Divorced/widowed (ref: married)	0.6009	0.2861–1.2624	0.1787
Single (ref: married)	0.7694	0.3471–1.7056	0.5187
Experience ≤10 y (ref: ≥16 y)	2.9092	0.8163–10.3677	0.0996
Experience 11–15 y (ref: ≥16 y)	0.8369	0.3677–1.9044	0.6712
Chronic disease: yes (ref: no)	0.6798	0.4003–1.1544	0.1532
Database access: yes (ref: no)	0.6562	0.3404–1.2650	0.2084
Database access: unsure (ref: no)	0.9824	0.4347–2.2200	0.9659
Internet use 3–6 h/day (ref: ≤3 h)	2.0437	1.1467–3.6423	0.0153
Internet use >6 h/day (ref: ≤3 h)	1.1743	0.6367–2.1658	0.6070
Online health search daily/several times weekly (ref: rarely/never)	2.3030	0.9923–5.3453	0.0522
Online health search weekly/several times monthly (ref: rarely/never)	1.9262	0.7077–5.2430	0.1994
eHEALS total score (per point)	0.9875	0.9599–1.0158	0.3825

**Table 8:** Multivariable linear regression analysis of factors associated with e-health literacy (HC3-robust standard errors).

Predictor	B	95% CI	*p* Value
Age (per year)	−0.0640	−0.2252 to 0.0973	0.4367
Male (ref: female)	−1.3625	−3.8325 to 1.1075	0.2796
Nursing/midwifery (ref: specialist physician)	0.7205	−1.6857 to 3.1266	0.5573
General practitioner (ref: specialist physician)	−0.6104	−3.0390 to 1.8182	0.6223
Divorced/widowed (ref: married)	−4.4087	−7.4003 to −1.4171	0.0039
Single (ref: married)	0.4158	−2.2706 to 3.1023	0.7616
Experience ≤ 10 y (ref: ≥16 y)	0.1633	−3.5133 to 3.8400	0.9306
Experience 11–15 y (ref: ≥16 y)	0.3197	−2.3853 to 3.0248	0.8168
Chronic disease: yes (ref: no)	1.4765	−0.2686 to 3.2216	0.0973
Database access: yes (ref: no)	−0.9641	−3.5626 to 1.6345	0.4671
Database access: unsure (ref: no)	−6.1805	−8.9255 to −3.4356	<0.0001
Internet use 3–6 h/day (ref: ≤3 h)	1.5646	−0.5281 to 3.6572	0.1428
Internet use >6 h/day (ref: ≤3 h)	−1.0832	−3.7584 to 1.5921	0.4275
Online health search daily/several times weekly (ref: rarely/never)	0.2319	−2.2204 to 2.6842	0.8530
Online health search weekly/several times monthly (ref: rarely/never)	−1.9775	−5.2953 to 1.3402	0.2427
Read online health information: sometimes (ref: never/rarely)	−0.9595	−3.5256 to 1.6066	0.4636
Read online health information: often/always (ref: never/rarely)	2.6264	0.5112 to 4.7416	0.0149
Preferred health website/social media source: yes (ref: no)	1.5233	−0.7507 to 3.7973	0.1892
LLM use: yes (ref: no)	−1.0560	−2.9840 to 0.8720	0.2831

**Table 9:** Comparison of LLM use and e-health literacy across professional groups.

Profession	n	LLM Use, n (%)	eHEALS, Mean ± SD
Midwife	36	33 (91.7)	27.75 ± 8.10
Nurse	124	87 (70.2)	29.09 ± 7.00
General practitioner	108	69 (63.9)	28.18 ± 10.25
Specialist physician	112	89 (79.5)	28.34 ± 10.30

**Table 10:** Profession-based subgroup analysis of LLM use and e-health literacy.

Outcome	Factor (Reference)	aOR	95% CI	*p*
Frequent LLM use	Male vs. female	4.0796	2.0030–8.3087	0.0001
Frequent LLM use	Age, per year	0.9542	0.9190–0.9908	0.0148
Frequent LLM use	Institutional database: not sure vs. no	0.3826	0.1622–0.9023	0.0286
Frequent LLM use	>6 h/day internet vs. ≤3 h/day	2.4239	1.1166–5.2618	0.0250
Frequent LLM use	Online health seeking: weekly/several times monthly vs. rarely/never	4.6444	1.5374–14.0290	0.0065
Satisfied/very satisfied	Male vs. female	1.8138	1.0738–3.0637	0.0260
Satisfied/very satisfied	Specialist physician vs. midwife	0.3937	0.1624–0.9545	0.0391
Satisfied/very satisfied	Institutional database: not sure vs. no	0.4455	0.2219–0.8943	0.0230
Satisfied/very satisfied	Online health seeking: weekly/several times monthly vs. rarely/never	2.7533	1.0989–6.8988	0.0307
Accurate/reliable: Yes	General practitioner vs. midwife	0.2624	0.1011–0.6807	0.0059
Accurate/reliable: Yes	Specialist physician vs. midwife	0.1679	0.0626–0.4506	0.0004
Accurate/reliable: Yes	Online health seeking: daily/several times weekly vs. rarely/never	0.3789	0.1682–0.8535	0.0192

Note: Only statistically significant adjusted associations are displayed for compactness. All models additionally included chronic disease and total eHEALS score; non-significant covariates were retained in model estimation. The frequency analysis included LLM users only (n = 278). Satisfaction and reliability analyses used the responses recorded for the full sample (n = 380).

## Data Availability

The data presented in this study are available on request from the corresponding author. The data are not publicly available due to privacy and ethical restrictions.
